# Female Saudi College students' e-learning experience amidst COVID-19 pandemic: An investigation and analysis

**DOI:** 10.1016/j.heliyon.2022.e12768

**Published:** 2022-12-31

**Authors:** Hadil Shaiba, Maya John, Souham Meshoul

**Affiliations:** a Department of Computer Sciences, College of Computer and Information Sciences, Princess Nourah bint Abdulrahman University, P.O. Box 84428, Riyadh, 11671, Saudi Arabia; b Independent Research, Kerala, India; c Department of Information Technology, College of Computer and Information Sciences, Princess Nourah bint Abdulrahman University, P.O. Box 84428, Riyadh, 11671, Saudi Arabia

**Keywords:** E-learning, COVID-19, ICT, Statistical analysis, Online transition, Student perspective

## Abstract

Emergency remote teaching in the immediate wake of the COVID-19 pandemic has created a challenging situation for both students and teachers. The purpose of this research is to identify the perceptions and challenges that university students faced during online classes in a women only university in Saudi Arabia. Data was collected by circulating Google forms among students from different colleges, and a total of 542 students submitted their responses. Apart from gathering the personal information of participants, the survey also collected information on aspects such as educational, financial, internet connectivity and volunteering/donations. Chi-squared test was used to determine whether there was a significant difference in opinion between different groups of students on various questions. Stress was identified as the most prevalent issue among students. Students were found to be stressed regardless of their college of study or age. In comparison to others, younger students and students from financially disadvantaged families faced more difficulties. In terms of remote practical class satisfaction, health/medical stream students were the most dissatisfied group. They also faced more difficulties than students from other colleges. The analysis results show that problems such as stress, poor internet connectivity, the need for technical support, a lack of proper interaction with faculty, a lack of proper academic advising, a lack of proper study space at home etc. must be addressed in order to improve the effectiveness of online classes. This paper also includes recommendations for resolving the various issues that students face.

## 1 Introduction

The COVID-19 lockdown has resulted in the closure of numerous educational institutions worldwide, resulting in an abrupt and rapid shift to online learning, teaching, and assessment (LTA) also known as emergency remote LTA. Some refer to the abrupt change as Emergency Remote Teaching (ERT). ERT is defined as an unexpected transition from physically taught courses to virtual learning, as opposed to online learning, which follows a predetermined and planned model [1]. As a result of the abrupt migration, faculty, staff, and students have faced numerous challenges. During the pandemic, countries such as Saudi Arabia, Egypt, France, the United States of America, Italy, Japan, China etc. used the internet and television to deliver lessons to students. The United Arab Emirates (UAE) mobilized government agencies and stakeholders to raise awareness about online classes and communicate with students, parents, and teachers about them. Countries such as Saudi Arabia, for example, have used Twitter to disseminate information about online education.

According to students enrolled in chemistry courses at the University of British Columbia's Okanagan campus, emergency remote learning has increased their stress and anxiety levels, caused scheduling issues, resulted in a communication gap with faculty, and decreased their motivation to continue their studies [2]. Respondents stated that combining recorded lectures with interactive online classes improved their learning experience. Students in underdeveloped countries such as Ghana lacked the motivation to attend online classes due to factors such as poor internet connections and lack of knowledge about e-learning platforms. Students who participated in a Ghana University study believe that motivation levels could be increased if, among other things, there was consistency in the online platform used for learning, the use of personal learning gadgets, and low-cost access to high-speed internet [3]. Giovannella et al. conducted research on school teachers' perceptions of ERT in Italy two months after the lockdown began [4]. Teachers reported difficulty in managing their time as their workload increased. Their increased trust in digital teaching approaches resulted in a shift in their attitude toward the teaching process. They believed that blended teaching should be used instead of traditional teaching and that future instructors should be trained in the use of e-learning tools.

In a study of 100 Saudi Arabian students studying English as a foreign language, the students stated that their main barriers to e-learning were lack of proper technical knowledge, lack of prior experience with an e-learning system, lack of motivation, personal cognitions such as lack of confidence in using the new system, and technical glitches such as lack of high bandwidth and poor internet connectivity [5]. The authors believed that hosting webinars and creating demonstrations about e-learning would help students feel more at ease with the new system. A study based on Umm Al-Qura University students in Saudi Arabia analyzed the impact of COVID-19 pandemic on society and education [6]. The results showed that the impact on the societal aspect was more prominent when compared to the educational aspect. A countrywide survey was conducted among dental students in Saudi Arabia to study the psychological impact of COVID-19 [7]. A significant difference was observed in terms of university and gender. High levels of stress, depression and anxiety were reported among students. An opinion poll conducted among university students in KSA regarding their academic perceptions and course satisfaction during online classes reported that workload and inadequate technical support led to negative perceptions which in turn resulted in low levels of course satisfaction [8]. A study was conducted among engineering students in a university in KSA to gather details regarding the experience and perceptions regarding the Blackboard E-learning platform [9]. A significant difference was observed between male/female students and students from different disciplines in engineering with regard to the effectiveness of the e-learning platform. The survey results of female students from the College of Language and Translation at King Saud University showed that many students were happy with online learning [10]. According to them the major drawback of remote learning were lack of self-motivation, technical glitches and problems due to the absence of face-to-face communication.

A study was conducted to analyze the problems faced by female students enrolled for undergraduate courses in a computer science college in Saudi Arabia [11]. The students were also asked to give recommendations on how the online learning experience can be made more fruitful. The major challenges faced by students were technical issues, inadequate interaction with faculty, lack of focus, absence of systematic schedule etc. 281 students from health colleges in various universities in Saudi Arabia participated in an online study to help authorities gather information regarding their perceptions about online learning during the time of pandemic [12]. The students shared their opinions regarding online teaching and learning process, technical support during learning, how the instructor can help in improving the learning experience and reasons for endorsing/not endorsing online learning in future. A cross-sectional study conducted among undergraduate students in the medical department at King Saud University, KSA analyzed various aspects regarding stress and measures taken by students to tide over the stress [13]. Stress was analyzed in terms of gender, academic variables, psychological variables etc. A survey among different types of health stream students such as medical, applied health and dentistry in different universities in Saudi Arabia studied the effectiveness of emergency remote learning [14]. A significant difference in satisfaction level was observed between male and female students. Students from the medical stream were least satisfied with online classes. Irrespective of gender and stream of study students preferred blended learning over online learning.

In the Kingdom of Saudi Arabia (KSA), the Ministry of Education has announced the temporary suspension of studies in all regions of the Kingdom on Monday, March 9th, 2020, and the activation of distance learning at all educational levels. Both public and private schools and institutions were affected by the decision. On March 18, 2020, the Ministry of Education instructed schools and colleges to postpone final exams and replace them with alternative arrangements that do not impact academic outcomes. On August 15, 2020, the Ministry announced that the first seven weeks of the new academic year would resume distantly. However, due to the pandemic situation, distance education has resumed until the end of the 2021 academic year [15].

This paper examines the university students opinions and challenges encountered during the remote learning phase due to COVID-19. The data for the analysis was gathered by distributing a questionnaire to female college students in the KSA. This type of analysis assists policymakers in better preparing for similar situations that may arise in the future. In this study, questions were based on aspects such as opinions about online learning, study device, study space, internet connectivity, teaching process, additional support needed, financial aspect and volunteering/donation. To the best of our knowledge, there are no similar studies based on female students in Saudi Arabia which take into consideration such diverse aspects and different majors of study.

The remainder of the paper is structured as follows: Section 2 focuses on the study objectives. Section 3 describes our data and methodology. Section 4 presents the results, while Section 5 discusses the results, implications and limitations of the study. Finally, section 6 draws the conclusions.

## 2 Study objectives

The overall objective of this study is to explore the students' perspectives and challenges as a result of emergency remote teaching. This form of analysis is critical for enhancing the quality of the teaching and learning process. The data was collected to analyze the following aspects.

1. Opinion about online learning, teaching and need for further assistance
2. Identify group of students who faced more challenges than others
3. Effect of the pandemic on the financial situation and related aspects
4. Study devices and online platforms
5. The student's willingness to assist other students by volunteering and donating
6. Identify the main problems faced by students

There is a general notion that computer stream studies might find it easier to shift to remote learning as they are more likely to be technically superior than others. A study based on Ukraine university students reported that students from certain disciplines found the transition to remote learning relatively smoother than others [16]. Several studies reported that students prefer conventional teaching mode over online learning [17]. In several countries, students enrolled in medical courses suffered the most from remote practical classes during the current pandemic [18,19]. Students’ lack of effective interaction with peers/teachers and lack of proper digital devices due to financial constraints adversely affected the online learning [11]. On the basis of analysis of various related studies from around the world, several questions were formulated to analyze the study objectives.

In order to identify the category of students who faced more challenges, the following research questions were analyzed.

1. Did students in computer science colleges find it easier to transition to online learning than students in other fields?
2. When compared to students in the humanities stream, do science college students favor traditional mode of learning over online learning?
3. Are students in health/medical colleges more likely to be dissatisfied with remote practical classes?
4. Are computer college students more effective at resolving hardware or software difficulties, and do they believe online classes are running smoothly?
5. Did students seek remote tutoring as a result of difficulties following online classes and a lack of engagement with faculty?

The financial aspect based analysis was conducted by analyzing the following research questions.

1. Are students with financial troubles more likely to struggle than others?
2. Did the economic situation due to COVID-19 affect the learning environment?

## 3 Data and methods

In this section, we explain the methodology we have adopted and describe in detail the main processes performed during this study.

**Fig. 1:**
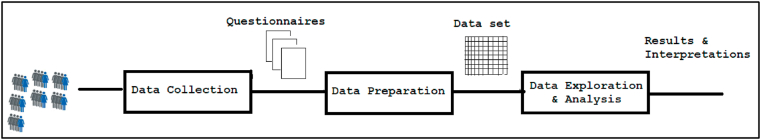
An overview of the methodology.

The methodology adopted to conduct this study follows a multi-stage process that can be summarized as shown in Fig. 1. As illustrated in Fig. 1, the study includes three main phases which are: data collection, data preparation, and data exploration and analysis.

### 3.1 Data collection

The data was collected from students during the period from August 2021 to September 2021. E-mail regarding the survey was circulated to authorities from different colleges in the university who in turn shared it with the students. The online survey was conducted using Google forms. The study was conducted according to the guidelines of the Declaration of Helsinki and approved by the Institutional Review Board of Princess Nourah bint Abdulrahman University (protocol code 21–0331 and date of approval August 14, 2021). Students’ consent to fill out the questionnaire for the purpose of this research was taken. The respondents included 542 female students from 16 different colleges. The respondents were aged between 17 and 47 years.

### 3.2 Questionnaire design

The questionnaire was designed in a way to meet the research objectives and to ensure clarity, brevity and completeness. The introductory part of the questionnaire included basic information about the respondents such as age, major, college and city of residence. The body of the questionnaire included a set of Likert scale, as listed in Table 1. In addition to the questions in Table 1, the questionnaire contained a question as shown in Table 2 that allowed the student to choose one or more types of challenges she had encountered throughout her transition to online learning. Each student was able to select one or more problems faced (if any existed). Questions on the following aspects were also included in the survey.

1. The type of device used for study.
2. Student's opinion about easiest and most difficult online learning platforms.
3. Student's opinion about remote practical classes.

**Table 1:** A Set of questions in survey with Likert scale.

Statement	Type of Answer
**Q1:** It was easy to switch to online learning	**Three level Likert item:** Score based on (Table 3)
**Q2:** Prefer traditional learning method to online learning	
**Q3:** Remote lectures were carried out smoothly	
**Q4:** Student owns device used for study	**Three level Likert item:** Score (Table 4)
**Q5:** Adequate study space at home	**Three level Likert item:** Score (Table 5)
**Q6:** Weak internet connection affected studies	**Three level Likert item:** Score (Table 3)
**Q7:** Good interaction with faculty	**Three level Likert item:** Score (Table 3)
**Q8:** Remote advising was effective	
**Q9:** Faced technical issues	
**Q10:** Felt like talking to counsellor to deal with social/psychological issues	**Three level Likert item:** Score (Table 3)
**Q11:** Have you joined/needed remote tutoring	
**Q12:** Felt need for technical support	
**Q13:** Pandemic adversely affected financial situation of family	**Three level Likert item:** Score (Table 3)
**Q14:** Pandemic negatively affected the eating habits	
**Q15:** Willingness to volunteer as tutor	**Three level Likert item:** Score (Table 3)
**Q16:** Willingness to donate book, devices, study space	**Three level Likert item:** Score (Table 3)

**Table 2:** Issues faced during the online learning shift due to COVID-19.

Type of issue/issues faced during the online shift	Type of Answer
Stress, depression, and anxiety	Yes/No
Lack of a special device for studying	Yes/No
Lack of a suitable environment for study	Yes/No
Poor internet connection	Yes/No
Inability to follow online lectures	Yes/No
Difficulty communicating with the teacher	Yes/No
Difficulty finding academic advising	Yes/No
Difficulty finding psychological support	Yes/No
Technical difficulties	Yes/No
Difficulty communicating with other students	Yes/No
None of the above	Yes/No

**Table 3:** A three level-Likert score assigned to responses of students with different views.

Yes (Group 3)	Somewhat (Group 2)	No (Group 1)
3	2	1

### 3.3 Data preparation

The major, college, city of residence features were manually checked and similar words were unified. The survey was in Arabic and was translated into English. In the data preparation phase, a three level-Likert item has been adopted to scale the responses we got from students for different questions. Points were assigned corresponding to responses mentioned by students are shown in Tables 3–5.

**Table 4:** A three level-Likert score assigned to responses based on students’ ownership of a study device.

Others (Group 1)	Family member (Group 2)	Self (Group 3)
1	2	3

**Table 5:** A three level-Likert score assigned to students’ opinion about the study space.

Comfortable (Group 3)	Inadequate (Group 2)	No study space (Group 1)
3	2	1

The score points were assigned as shown in Table 3, except for the questions that are related to the study space adequacy and device used for study. If a student owned a study device, a score of 3 was assigned, a score of 2 if it belonged to a family member and a score of 1 was assigned otherwise, as shown in Table 4. The student's opinion about the availability of study space at home was encoded as shown in Table 5.

### 3.4 Data exploration and analysis

The question related to eating habits was not considered for further analysis as several students did not respond to the question. Exploratory factor analysis (EFA) was performed on data with regard to questions mentioned in Table 1 except the one related with eating habit. Before EFA is performed, the adequacy of sample size and data is to be checked using Kaiser-Meyer-Olkin (KMO) and Bartlett's Test of Sphericity [20]. The EFA was implemented with rotation method set to Promax as the variables considered may have some dependence. Various representations have been derived throughout the data analysis process. Summaries in percentages have been calculated as a numerical representation. In order to identify whether there is significant difference in the opinion between different groups of students, Chi-square test was carried out using R programming language. To determine the significance of the chi-square scores, p-values were calculated. A p-value of p < 0.05 was considered significant.

## 4 Results

The results of the survey and the analysis carried out is described in this section.

### 4.1 Opinion overview

Table 6 shows an overview of the students’ views about different aspects of the online shift.

**Table 6:** An overview of the students’ opinion.

Statement	Group3	Group2	Group1
**Q1:** It was easy to switch to online learning	32.84%	37.08%	30.07%
**Q2:** Prefer traditional learning method to online learning	54.61%	23.06%	22.32%
**Q3:** Remote lectures were carried out smoothly	43.54%	36.35%	20.11%
**Q4:** Student owns device used for study	89.30%	9.96%	0.55%
**Q5:** Adequate study space at home	53.14%	32.29%	14.39%
**Q6:** Weak internet connection affected studies	46.13%	26.01%	27.86%
**Q7:** Good interaction with faculty	35.42%	34.69%	29.70%
**Q8:** Remote advising was effective	32.66%	37.45%	29.89%
**Q9:** Faced technical issues	27.12%	23.62%	49.26%
**Q10:** Felt like talking to counsellor to deal with social/psychological issues	36.53%	14.39%	49.08%
**Q11:** Have you joined/needed remote tutoring	38.56%	7.56%	53.87%
**Q12:** Felt need for technical support	37.64%	19.74%	42.62%
**Q13:** Pandemic adversely affected financial situation of family	19.74%	23.25%	57.01%
**Q14:** Pandemic negatively affected the eating habits	14.76%	11.99%	56.27%
**Q15:** Willingness to volunteer as tutor	22.51%	28.97%	48.52%
**Q16:** Willing to donate book, devices, study space	41.33%	31.92%	26.75%

Table 6 shows that about only one-third of the respondents did not find it really easy to switch to online learning. This may be due to the fact that students are more tech-savvy now-a-days. More than half of the respondents prefer traditional learning and less than 20% found that lectures were not carried out smoothly. The majority of students (89.30%) carried out their studies using their own personal devices. Just over half of the students were comfortable with the study space they had at home. According to the findings, 46.13% had a poor internet connection, 26.01% faced some internet connection issues, and 27.86% did not face any internet connection challenges. It can be observed from Table 6 that students had similar feelings regarding good interactions with faculty and the usefulness of remote advising. Around 50% of the students did not face technical issues. Results show that a significant number of students representing 37.64% required technical assistance, 36.53% desired to talk with a counsellor to deal with social or psychological issues, and 38.56% had joined or required tutoring. These difficulties are researched further in order to help in developing reliable solutions and making better decisions. In the case of around 43% of the students, COVID-19 negatively affected the financial situation of their families. 27% of the students responded that their eating habits were negatively affected due to the pandemic. Students prefer donating resources such as books and electronic devices over volunteering to tutor others with a percentage of 41.33% versus 22.51%. Students can be encouraged to donate items like mobile phones, books, laptops etc. and serve as tutors for other students.

### 4.2 Exploratory Factor Analysis

Exploratory Factor Analysis was conducted for questions mentioned in Table 1 with exception of question related to eating habit as EFA cannot be performed on variables with missing values. EFA was performed on the responses of 542 students with Promax as the rotation function. The sample adequacy test KMO yielded a value of 0.84. The value of KMO ranges from 0 to 1 and value less than 0.6 indicates that set of variables are not suitable for factor analysis [21]. The result of Bartlett's Test of Sphericity is tabulated in Table 7. The results indicate that data is appropriate to perform EFA.

**Table 7:** Bartlett's test of sphericity.

chisq	1850.792
p.value	7.06e-317
df	105

It is evident from Table 8 that the first factor is associated with teaching/learning process, factor two is related to internet and technical aspects, factor three deals with financial related aspects and fourth factor deals with students’ opinion about volunteering and donation. The correlation between the factors are shown in Table 9.

**Table 8:** Result of EFA analysis.

Feature	Factor1	Factor2	Factor3	Factor4
Remote shift easy	0.756			
Traditional learning over distance learning	−0.502			
Remote lectures were smooth	0.767			
Good remote interaction with faculty	0.813			
Need for remote tutoring	−0.296			
Opinion about remote advising	0.408			
Weak internet connection		0.490		
Encountered hardware software issues		0.793		
Need for technical support		0.826		
COVID negative effect on economy			0.653	
Device owner			−0.362	
Study space availability			−0.500	
Need for social psychological counsellor			0.210	
Volunteer as tutor				0.353
Willing to donate				0.746
Percentage of variance (%)	16.67	10.87	5.93	4.67

**Table 9:** Correlation between factors.

	Factor1	Factor2	Factor3	Factor4
Factor1	1	–	–	–
Factor2	−0.5405	1	–	–
Factor3	−0.0093	−0.065	1	–
Factor4	−0.5599	0.635	−0.0281	1

### 4.3 Major issues

**Fig. 2:**
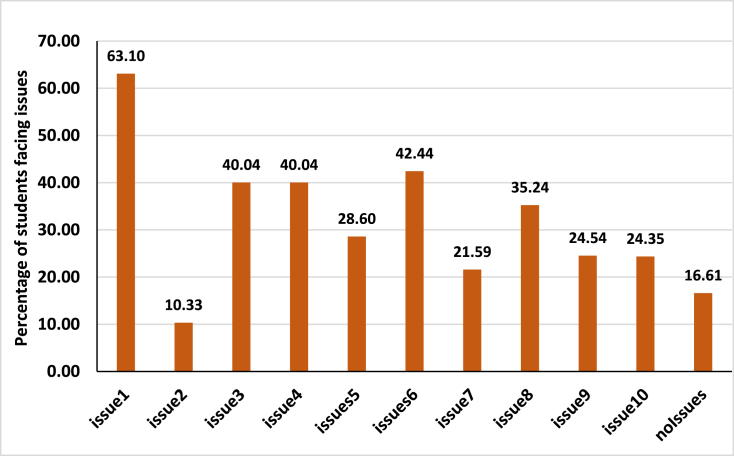
Percentage of students facing each type of problem.

For the sake of diagrammatic representation, the problems faced by students are encoded in Table 10. Fig. 2 shows the percentage of students facing each type of issues.

**Table 10:** Encoding of the problems faced by students.

Encoding	Problem
Issue1	Stress, depression and anxiety
Issue 2	Lack of a special apparatus for studying
Issue 3	Lack of a suitable environment for study
Issue 4	Poor internet connection
Issue 5	Inability to follow online lectures
Issue 6	Difficulty communicating with the teacher
Issue 7	Difficulty finding academic advising
Issue 8	Difficulty finding psychological support
Issue 9	Technical difficulties
Issue 10	Difficulty communicating with other students
None	None of the above

Fig. 2 shows that stress, depression and anxiety, lack of a suitable environment for study, poor internet connection, difficulty communicating with the teacher, and difficulty finding psychological support are among the five main issues students are facing. As the major issue the students faced was stress, we further analyzed the factors which might have resulted in stress.

#### 4.3.1 What are the elements that contribute to students' stress?

To study the factors that are significantly related to students being stressed, we performed the Chi-squared test between the stressed students and others using factors such as having adequate study space, having weak internet connection, etc. as shown in Table 11.

**Table 11:** Analysis of dependence between students stress and potential factors.

Factors	$\chi^{2}$	p-value
Q5:Adequate Study space	51.511	**6.526e-12**
Q3:Remote lecture smooth	66.696	**3.289e-15**
Q7:Good Interaction with faculty	55.944	**7.11e-13**
Q4:Device Ownership	4.3136	0.1157
Q6:Weak internet connection	54.997	**1.142e-12**
Q13:Financial situation	7.4444	**0.02418**
Q14:Change in eating habits	15.777	**0.0003751**
Q10:Need For Social Psychological Counsellor	106.11	**2.2e-16**
Q8:Effective remote advising	39.013	**3.376e-09**
Q12:Need for technical support	29.317	**4.305e-07**

p-value less than 0.05 (indicates significant difference) is reported in bold font.

**Fig. 3:**
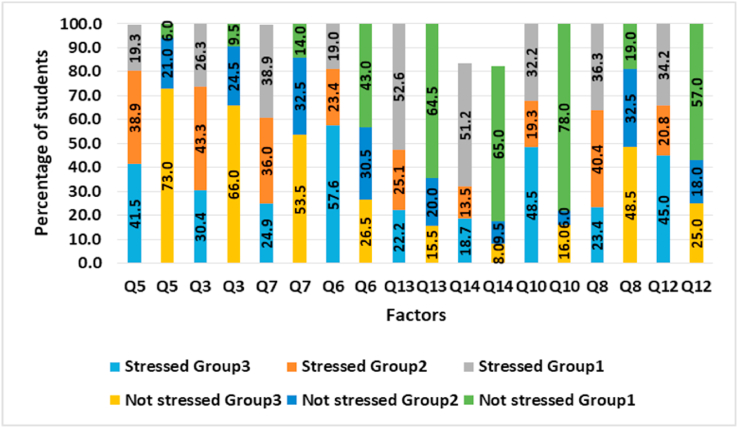
Overview of percentage of students facing problems.

Table 11 shows that many factors have a significant dependence on students being stressed. We can see from Table 11 that issues like not having adequate study space, not having smooth remote lectures, not having good interaction with faculty members, having a weak internet connection, being financially affected by the pandemic, change in eating habits, needing to talk to a counsellor to deal with social/psychological issues, ineffective remote advising, and need for technical support showed a significant difference between the stressed students and the rest. Fig. 3 pictorially gives an overview of the student responses with regard to different levels of stress. Group3, Group2, and Group1 correspond to responses yes, somewhat and no for the respective question in the survey. It is clear from the figure that stressed students face more issues than others.

#### 4.3.2 Did students experience the same challenges with remote education regardless of their college of study?

**Fig. 4:**
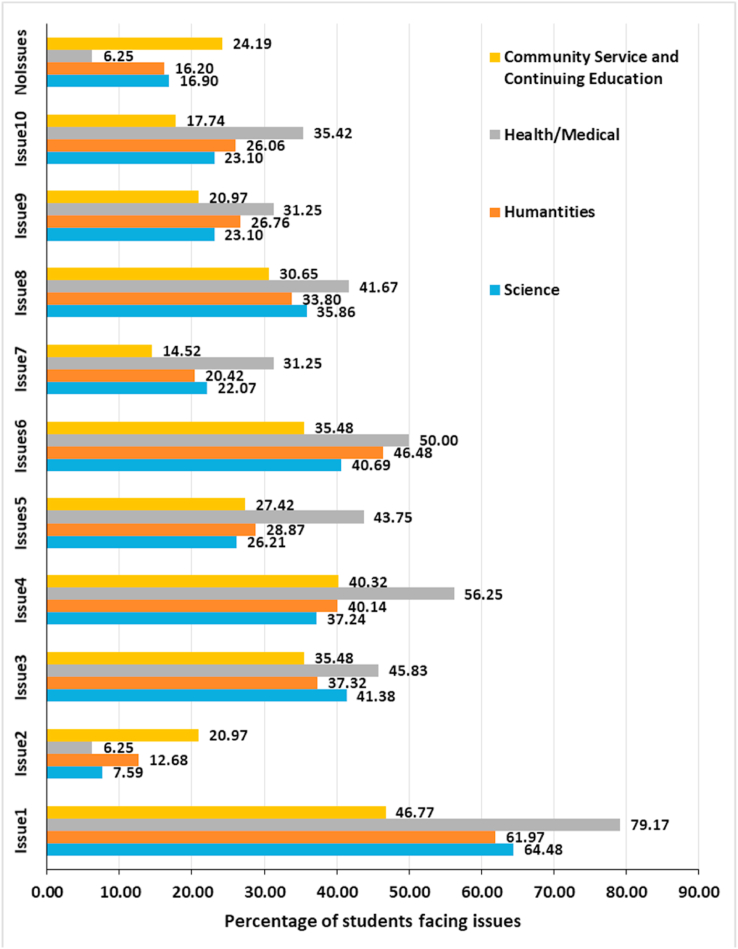
Percentage of students facing each problem based on the student's college of study.

Corresponding to each college, Fig. 4 shows the percentage of students facing each problem. Issues listed in Table 10 are considered for this Figure. The colleges were grouped into four groups namely: Science, Health Science, Humanities, and Community Service and Continuing Education. It is clear from the figure that regardless of college of study, stress is the most common issue faced by students. It is obvious from Fig. 4 that students from Health Sciences fields faced more issues than students from other fields. Further investigation need to be carried out to understand why Health Science students’ face more issues than others.

### 4.4 Study equipment, online platform

The students were asked about the device they used for studying during online classes. 30% of students depended on mobile and tablets only for studying while 18% used desktop/laptop only for studying. It is to be noted that using mobile phone only for online learning reduces the ease of learning [22]. Students' opinion was also collected regarding which online platform was easiest and most difficult to use. Nearly 65% of the students responded that they were most comfortable with Microsoft Teams. 22% of the students felt that Blackboard was the easiest platform to use and 12% found Zoom relatively easy to use. Around 50% of the students were of the opinion that they were least comfortable with Blackboard. 18% of the respondents found Zoom most difficult to use. It is to be noted that Microsoft Teams and Blackboard are the most commonly used platforms in the university under study as it is subscribed to Microsoft Teams and Blackboard and so many of their functionalities are freely available for faculties and students. This might be the reason why the university students are more familiar with Microsoft Teams when compared to Zoom which requires subscription to get full benefit of its functionalities. Blackboard is mostly used to download the materials of the courses and submit student's grades and assignments although some faculties conduct online lectures through it.

### 4.5 Students category and challenges

This section deals with the analysis of the difference between various categories of students with regard to online learning experiences and challenges.

#### 4.5.1 Did students in computer science colleges find it easier to shift to online learning than students in other fields?

The opinion of students regarding the ease of switching to online classes is summarized in Table 12.

**Table 12:** Students’ opinion about the ease of switching to online learning.

Opinion	Computer College	All Colleges
Yes	38.89%	30.26%
Somewhat	38.89%	36.32%
No	22.22%	33.42%

Chi-Squared test has been conducted where the dependence between the type of college and opinion regarding online learning has been considered. As a first step, we considered Computer College versus all Colleges and as a second step Computer College versus each College separately.

The dependence test assumes the following Null and alternative hypotheses.

H0 the two variables (college and opinion) are independent.

Ha the two variables (college and opinion) are dependent.

Then, we performed the Chi-squared test. In this case, we found that the value of the test statistic is as follows:

$$\chi^{2}=7.51254\;and\;p\_value=0.02337$$

As the p_value is less than the significance level which is 0.05 (for 95% confidence level) this means that the opinion of students depends on the type of college. This confirms the hypothesis mentioned above. This is due to the fact that computer science students are technically superior to others.

We further investigated the results by performing a Chi-squared test between the Computer Science College and every other College to study the dependence between the type of college and the opinion of students. The values of the test statistics are shown in Table 13.

**Table 13:** The values of the test statistics of the Computer Science College with each College.

College	$\chi^{2}$	p-value
Health/Medical	8.7299	**0.01272**
Science	7.0941	**0.02881**
Humanities	3.8278	0.1475
Deanship of community service	4.5905	0.1007

p-value less than 0.05 (indicates significant difference) is reported in bold font.

Comparing Computer College students to Health/Science College students, Table 13 indicates that there is a considerable difference in the ease of transitioning to online learning. This may be owing to the fact that practical classes for Computer Science students can be easily conducted remotely. In addition, online learning is inappropriate for Health and Science colleges because they require campus-based labs and practice. Comparing Computer College with Humanities and Deanship of Community Service, it was found that satisfaction levels are not reliant on the type of college, as the p-values were not statistically significant (p > 0.05). In contrast to Health and Science Colleges, online learning can serve the educational demands of students in Humanities and Community Service Colleges.

#### 4.5.2 When compared to students in the humanities stream, do science college students favor traditional mode of learning over online learning?

The opinion about students’ preference of traditional learning method over online learning is summarized in Table 14.

**Table 14:** The students’ opinion about preferring traditional learning over online learning.

Opinion	Science Stream	Humanities Stream
Yes	53.37%	56.99%
somewhat	25.56%	18.28%
No	21.07%	24.73%

The courses were classified into two streams named Science and Humanities and analysis was carried out. From Table 14, there seems to be no difference between Science and Humanities’ colleges when it comes to preferring traditional learning over remote learning. Both disciplines seem to prefer traditional methods. To confirm this, a chi-square test has been conducted. The value of the test statistic is as follows:

$$\chi^{2}=3.8367\;and\;p-value=0.1469$$

In this case, we can see that the p-value is greater than the significance level of 0.05 which means we can accept the hypothesis which indicates that there is no difference between the opinion of Science and Humanities students.

#### 4.5.3 Are students in health/medical colleges more likely to be dissatisfied with remote practical classes?

**Fig. 5:**
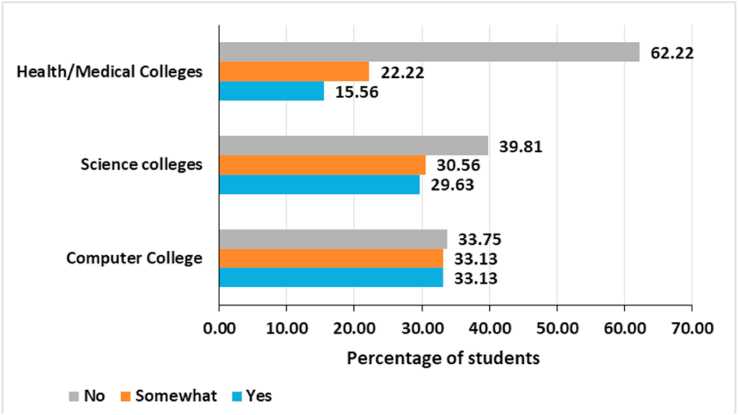
College-wise analysis of remote practical classes' satisfaction.

The analysis of remote practical class satisfaction for different colleges is shown in Fig. 5.

It is evident from Fig. 5 that students at Health/Medical Colleges and other Science Colleges (Engineering, Science, etc.) are more dissatisfied with remote practical classes. Comparatively higher levels of dissatisfaction were observed among the students studying health/medical courses.

#### 4.5.4 Are computer college students more effective at resolving hardware or software difficulties, and do they believe online classes are running smoothly?

Analysis was carried out to find out whether Computer College students were more effective in tackling hardware or software issues and felt online classes were running smoothly. Computer College students are expected to be superior in technical knowledge and hence are expected to have an edge over other students in the issues considered.

**Fig. 6:**
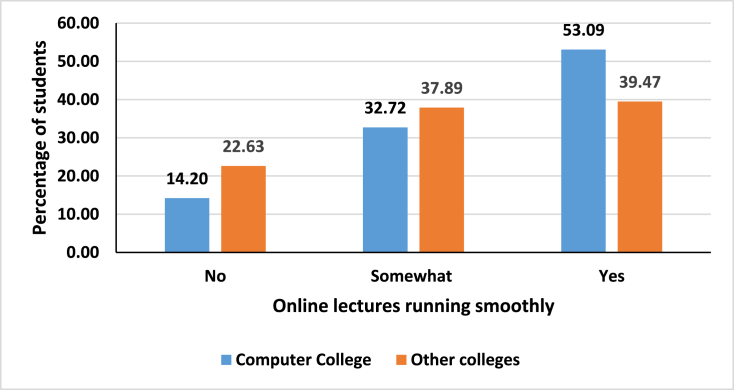
Opinion about online lecture running smoothly.

It can be observed from Fig. 6 that compared to other college students, Computer College students found that online classes are running more smoothly.

**Fig. 7:**
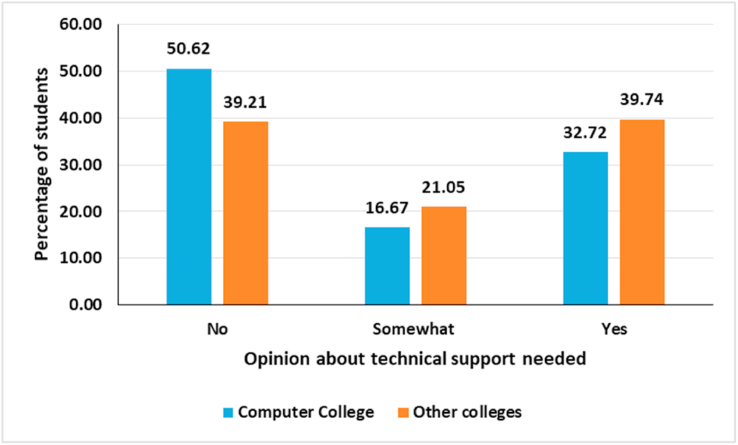
Opinion about technical support needed.

The students’ opinion regarding technical support needed is compared and pictorially depicted in Fig. 7. From the figure, it is evident that Computer Science students are slightly better at handling technical issues by themselves. The result of the Chi-square test performed is shown in Table 15.

**Table 15:** Chi-square test result.

Factors	$\chi^{2}$	p-value
Remote lecture smooth	4.3122	0.1158
Need for technical support	2.638	0.2674

Table 15 indicates that there is no statistically significant difference between the opinions of Computer College students and those of students from other disciplines regarding remote lecture delivery and the need for technical support.

#### 4.5.5 Did students seek remote tutoring as a result of difficulties following online classes and a lack of engagement with faculty?

To analyze whether the difficulty in following online classes and lack of interaction with faculty prompts students to join remote tutoring, the information collected is presented as shown in Table 16.

**Table 16:** Table showing percentage of students facing issues.

Issues		Difficulty in following online classes (Issue5)	
		Yes	No
Lack of interaction with faculty(Issue 6)	Yes	29.19%	25.84%
	No	13.40%	31.58%

As per the survey results, 209 students really needed/joined remote tutoring (38.5% of total students). We noticed that a large number of students needed or joined online tutoring, so we were interested to see the relationship between those who joined online tutoring and the issue of inability to follow online lectures and difficulty communicating with teachers. Nearly 70% of the students who really needed remote tutoring faced either issue 5 or issue 6 or both. Although 31.58% did not face issue 5 and 6, they still needed online tutoring. This might be an indication that remote learning require 1:1 teaching methods.

#### 4.5.6 Did younger students have more difficulties with distant learning than older students?

Analysis was carried out to find out whether younger students face more difficulties with remote learning when compared to other students. The average age of the respondents was 20.35 years, so we have grouped the students into two groups as follows: Group1: <21years and Group2> 20 years. Chi-squared test was performed to study whether there were significant differences between the two groups. Results are shown in Table 17.

**Table 17:** The values of the test statistics of the age group.

Issue	$\chi^{2}$	p-value
Q1:Remote shift	32.646	**8.147e-08**
QP:Remote practical class satisfying	10.368	**0.005606**
Q3:Remote lecture smooth	17.491	**0.0001592**
Q7:Interaction with faculty	7.1509	**0.028**
Q10:need For Social Psychological Counsellor	0.12666	0.9386
Q11:Need for remote tutoring	12.521	**0.001911**
Q8:Remote advising effectiveness	7.2403	**0.02678**
Q12:Need for technical support	5.5555	0.06218
I1:Faced stress, depression, anxiety	2.8538	0.09116
I5:Inability in following online classes	14.124	**0.0001711**
I10:Difficulty in communicating with other students	2.7109	0.09967

p-value less than 0.05 (indicates significant difference) is reported in bold font.

**Fig. 8:**
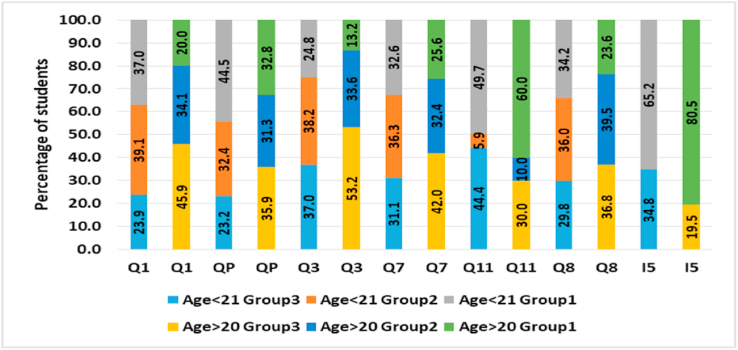
Comparison between age groups based on factors with significant difference.

**Fig. 9:**
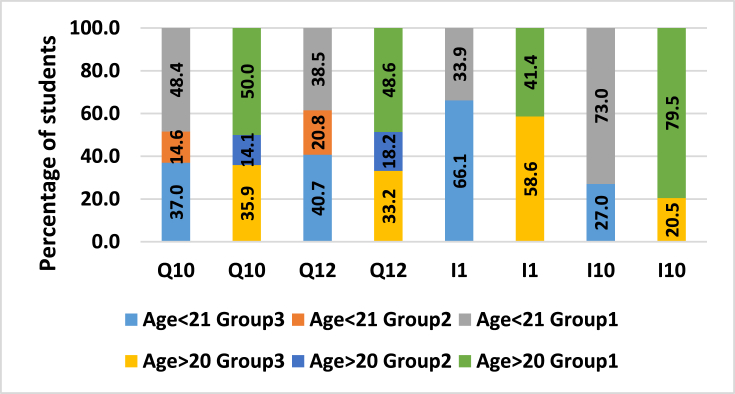
Comparison between age groups based on factors with no significant difference.

From the results shown in Table 17, we can infer that there is a significance difference between the two different age groups regarding remote shift, satisfaction about remote practical class, interaction with faculty, need for remote tutoring, effectiveness of remote advising and ability in following online classes. Plots were drawn to understand which group of students faced more problems. Fig. 8 shows the difference between two age group of students in terms of issues with significant difference. Fig. 9 depicts the percentage of students facing different issues with respect to issues where there is no significant difference between the age groups. It is evident from the aforementioned figures that students less age 21 years faced more problems when compared to rest of the students. Regardless of age, students were stressed and felt need for counsellors. Students lacked proper interaction with faculty but did not face much trouble in communicating with their peers.

### 4.6 Impact of COVID-19 on financial situation

The responses with regard to the impact of the financial situation on students are analyzed in this section.

#### 4.6.1 Are students with financial troubles more likely to struggle than others?

To study the differences between students being financially affected during the pandemic and those who are slightly affected and those who are not affected, we performed the Chi-squared test between different groups. The result of the analysis is tabulated in Table 18. It can be inferred from Table 18, the only significant difference between those who were mildly affected and those who are highly affected was with regard to inability to follow online lectures. Meanwhile significant differences between those who are not affected and those who are highly affected was observed in terms of that remote lecture going smoothly, the need for social or psychological counsellor, the student's opinion about remote advising, facing stress, depression, and anxiety, and the inability to follow online classes.

**Table 18:** Comparison of test statistics of the students who are financially affected by the pandemic with those who are slightly affected and those who are not affected.

Issues	Slightly affected		Not affected	
	$\chi^{2}$	p-value	$\chi^{2}$	p-value
Q3:Remote lecture smooth	5.0121	0.08159	14.545	**0.0006943**
Q7:Interaction with faculty	3.0604	0.2165	2.8243	0.2436
Q10:need For Social Psychological Counsellor	4.7993	0.09075	14.691	**0.0006454**
Q8:Opinion about remote advising	2.1858	0.3352	8.1948	**0.01662**
Issue1:Faced stress, depression, anxiety	0.099628	0.7523	4.9543	**0.02603**
Issue5:Inability in following online classes	8.2171	**0.00415**	24.377	**7.92e-07**
Issue10:Difficulty in communicating with other students	0.43402	0.51	0.92866	0.3352

p-value less than 0.05 (indicates significant difference) is reported in bold font.

**Fig. 10:**
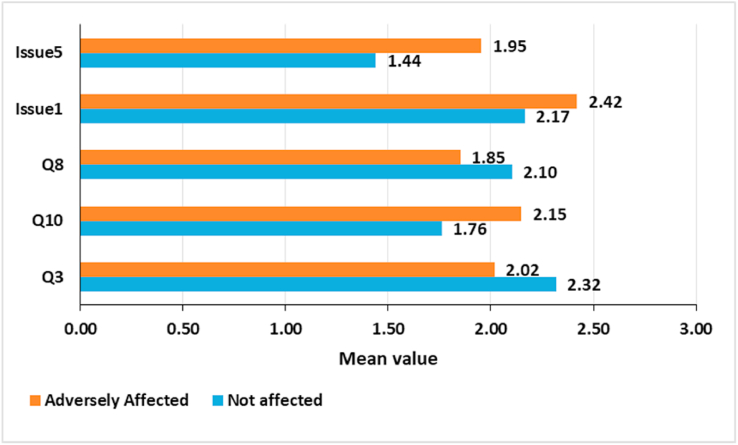
Comparison of financially adversely affected group and not affected group.

In order to understand which group of students suffered more problems, a graph was plotted to compare the extent of issues faced by the financially adversely affected students and those not affected. It is evident from the above Fig. 10 that students whose families were financially affected badly due to the pandemic suffered more problems.

#### 4.6.2 Did economic situation due to COVID-19 affect the learning environment?

**Fig. 11:**
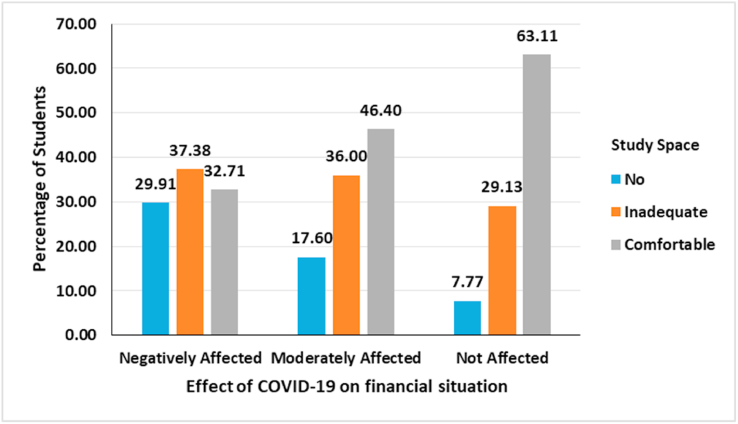
Relation between economic situation due to COVID-19 and having suitable study space.

**Fig. 12:**
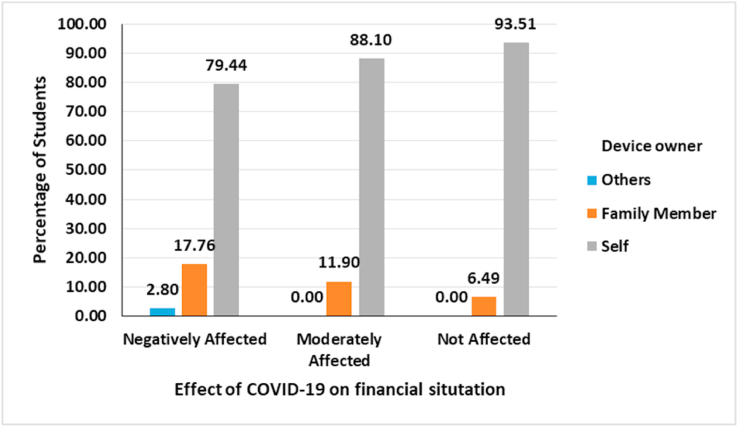
Relation between economic situation due to COVID-19 and owning a device.

Figs. 11 and 12 depicts the relationship between economic situation due to COVID-19 and having suitable study space and owning study device respectively. These types of plots help in analyzing whether financial situation has an impact on the students learning environment.

Owning a device and having an adequate study space is very important for students when studying remotely. It can be observed from Fig. 10 that students with financial difficulties had issues with having proper study space at home. It is evident from Fig. 12 that irrespective of the financial situation, vast majority of students had personal devices to carry out their academic activities.

## 5 Discussion

Around 55% of the students favoured traditional learning over online learning. Some earlier studies also showed that students preferred face-to-face learning over remote learning [23,24]. Similar to our findings, several studies also reported that students lacked proper internet connectivity during attending classes remotely [25,26]. Internet related issues will adversely affect the performance of students during online exams and in turn lead to psychological issues [27]. Nearly one-third of the students said that their eating habits were adversely affected due to the pandemic. Studies showed that stress and financial problems due to COVID-19 have contributed to eating disorders in youth [28]. A study based on students in West Bengal also reported that the major issues faced during online learning during lockdown were stress, poor internet connectivity and lack of proper study environment [29]. The results of the survey showed that health stream studies face relatively more issues compared to others. A previous work also reported that female students in medical stream are more stressed than others [30]. Responses from Turkish university students showed that Health stream students are more dissatisfied with remote learning when compared to the rest of the students [31]. The university students in Jordan were of the opinion that online classes are more suitable for Humanities courses than Science courses [32].

There was no significant difference between the science stream and the humanities stream with regard to the preferred mode of learning. Both groups preferred traditional learning over online learning. A study based on two universities in Romania also echoed the fact that students in general prefer traditional learning [33]. Health/medical stream students were least satisfied with the conduct of practical classes during remote classes during the pandemic. An earlier study based on medical college students observed that students experienced a significant imbalance between the theoretical and practical knowledge gained during online classes [34]. Several studies brought to light the fact that medical students suffered from the lack of practical classes and ineffective practical classes during remote learning [18,19]. Results of our study showed that over 50% of the students who felt the need for remote tutoring did not have proper communication with teachers. An earlier study reported that lack of interaction with faculty resulted in a poor learning experience, especially in the case of classes which can be rendered more effectively in traditional learning method. The students really missed traditional learning with regard to asking questions and receiving instantaneous responses from teachers [24]. Only around half of the respondents of our survey were fully satisfied with the study space at home. A study conducted by Raaper et al. also reported that students suffered due to the lack of proper study space. Their study also mentioned that certain parents did not understand the importance of providing proper study space to children at home during emergency remote learning [35].

### 5.1 Implications for practice

On the basis of students' views the following recommendations are put forward to enhance the students’ experience during online learning.

1. The results show that 60% of health college students were not satisfied with remote practical classes, while the level of dissatisfaction was less than 40% for science and computer college students. This stresses the need of incorporating technology to devise better methods to conduct experiments and enhance the clinical skills of students. Collaboration with colleges within the country and overseas will be useful in this regard [19].
2. The studies showed that nearly 70% of the respondents were not comfortable with using either Blackboard or Zoom. Hence students should be given training so that they can use them with ease.
3. In spite of the fact that almost all of the students lived in cities, nearly 50% of the students faced poor internet connectivity. This may be on account of the massive usage of internet [11]. Hence concerned parties (telecommunication companies) should take necessary measures to increase bandwidth and provide stable connection, in situations where the majority of students learn online.
4. Around 40% of the students felt that they needed technical support. Universities should provide adequate support to students to resolve technical issues [36].
5. Stress was the most common issue that the students faced. Regardless of the stream of study and age, stress was faced by the majority of the students. Over 35% of the students wished to talk with a counsellor due to social or psychological issues they faced. Online counselling may be adapted to deal with these types of issues. University of Salerno in Italy conducted online counselling through telephonic calls, psychological interviews etc. [37].
6. Nearly 40% of the students joined/needed extra tutoring. Around 55% of the students who needed/joined remote tutoring expressed the view that they did not have good interaction with faculty members. Faculty may encourage the usage of the hand raise feature, conduct online polls, use discussion forums etc. to enhance interaction with students during online classes. These should be linked to the learning activities and assessments so that students will be encouraged to use them [38].
7. About one-third of the students used only mobile phone/tablet for online learning. Such devices may not be much effective when compared to laptops/desktops. Decision makers should take further measures to provide students with laptops. Banks may provide loans to students to purchase laptops. Furthermore good mobile learning solutions will help in enhancing the effectiveness of learning using mobile phones. Hence it is essential for educators to comprehend how various mobile applications can aid in distance learning.
8. Many respondents were willing to volunteer in donation activities and tutoring. The concerned authorities should try to harness the spirit of students to engage in voluntary tutoring and donations to help other students.
9. The younger students, stressed students and financially adversely affected students did not find the remote advising system much effective. Technology assisted systems may be adopted to help students with remote academic advising [39].
10. It is crucial that universities adopt, recruit, and retain the most effective and successful ICT in education to improve the student's technical skills and learning experience and to optimize the teaching methods.

### 5.2 Future research directions and limitations of study

A major limitation of the study is that the study is restricted to the opinion of female students in a single university. Hence the results cannot be generalized as the experience of students may vary from university to university. The average age of the respondents was only 20 years, studies including more graduate students and higher degree students might help in getting a more unbiased overview of students’ perceptions/challenges during remote learning. Due lack of information regarding e-learning experience prior to the outbreak of COVID-19, no comparative study could be carried out between e-learning before and during the current pandemic. Studies may be conducted to analyze whether there has been significant improvement in online learning experience post-emergency online classes due to COVID-19. In this study, only the opinions of students are considered. An extensive study incorporating the views of other stakeholders like teachers, parents etc. will considerably help in providing students better e-learning experience in the future.

## 6 Conclusion

The study seeks to ascertain students' opinions and attitudes toward remote teaching. We examined the main issues that students were facing from various perspectives, which can help in decision making and in the improvement of online education. Although approximately 44% of students found remote lectures to be smooth, approximately 55% preferred traditional learning over distance learning. Traditional learning was preferred by both humanities and science students. When compared to the rest of the students, computer college students found it easier to transition to online learning. Students in the health/medical stream were the least satisfied with online practical classes. Around 40% of those polled felt they needed additional assistance, such as technical support, counselling, or remote tutoring. There was no significant difference between computer science students and the rest of the students in terms of the need for technical support and the delivery of remote lectures. Almost 70% of students who required/joined remote tutoring struggled with either following online classes or interacting with faculty, or both.

Almost all students had their own device for studying, but only half of them were satisfied with their home study space. Further investigation revealed that the majority of students from financially disadvantaged families as a result of COVID-19 were unsatisfied with their home study space. It was discovered that students whose families faced significant financial difficulties as a result of the pandemic faced more difficulties than others. Stress, depression, and anxiety were the most common problems reported by students, with approximately 60% of students experiencing them. The main sources of stress were identified as insufficient study space, a lack of proper interaction with faculty, a poor internet connection, financial difficulties, ineffective remote advising, a need for technical support, and social/psychological issues. Further investigation revealed that students faced numerous issues regardless of their field of study, with stress being the most prevalent issue across all disciplines. There was no significant difference between age groups in terms of stress, interaction with other students, need for technical support, and need for a counsellor. Students under the age of 21 had more difficulties than older students.

It is important to note that enabling the use of ICT technologies in education may help students and educators in many ways including the ease of access to online courses and materials, the utilization of flipped classrooms, and so on. The paper also puts forward recommendations which will help in solving the problems faced by students during remote learning. Solving the problems faced by students is vital in ensuring equity in education.

## Author contribution statement

Hadil Shaiba: Conceived and designed the experiments; Analyzed and interpreted the data; Contributed reagents, materials, analysis tools or data; Wrote the paper.

Maya John; Souham Meshoul: Conceived and designed the experiments; Performed the experiments; Analyzed and interpreted the data; Contributed reagents, materials, analysis tools or data; Wrote the paper.

## Funding statement

This work was supported by Princess Nourah bint Abdulrahman University (PNURSP2022R135).

## Data availability statement

The data that has been used is confidential.

## Declaration of interest’s statement

The authors declare no competing interests.
