# Supplementary material for: Female Saudi College students' e-learning experience amidst COVID-19 pandemic: An investigation and analysis
Source: Heliyon. 2022 Dec 31;9(1):e12768. doi: 10.1016/j.heliyon.2022.e12768 (PMC9803374; doi:10.1016/j.heliyon.2022.e12768)
Supplement: Multimedia component 1 [file mmc1.docx]

**Survey Questions**

1. Age of student:
2. Major of study:
3. College of study:
4. City of Residence:
5. Area of residence: Village/ City
6. It was easy to switch to online learning: Yes/No/Somewhat
7. Prefer traditional learning method to online learning : Yes/No/Somewhat
8. Remote lectures were carried out smoothly: Yes/No/Somewhat
9. Student owns device used for study: Own/Family Member/Others
10. Adequate study space at home: Comfortable/ Inadequate/No study space
11. Weak internet connection affected studies: Yes/No/Somewhat
12. Good interaction with faculty: Yes/No/Somewhat
13. Remote advising was effective: Yes/No/Somewhat
14. Faced technical issues: Yes/No/Somewhat
15. Felt like talking to counsellor to deal with social/ psychological issues: Yes/No/Somewhat
16. Have you joined/ needed remote tutoring: Yes/No/Somewhat
17. Felt need for technical support: Yes/No/Somewhat
18. Pandemic adversely affected financial situation of family: Yes/No/Somewhat
19. Pandemic negatively affected the eating habits: Yes/No/Somewhat
20. Willingness to volunteer as tutor: Yes/No/May be
21. Willingness to donate book, devices, study space: Yes/No/May be
22. Device used for study during online classes: Mobile Phone or Tablet only/ Laptop or Desktop only/ Mobile phone or tablet and laptop or desktop
23. Online platform easiest to use: Zoom/ Microsoft Teams/ Blackboard/ Others/Nothing
24. Online platform most difficult to use: Zoom/ Microsoft Teams/ Blackboard/ Others/Nothing
25. Remote practical classes satisfaction: Yes/No/Somewhat
26. Issues faced during classes:

- Stress, depression, and anxiety: Yes/No
- Lack of a special device for studying : Yes/No
- Lack of a suitable environment for study: Yes/No
- Poor internet connection: Yes/No
- Inability to follow online lectures: Yes/No
- Difficulty communicating with the teacher: Yes/No
- Difficulty finding academic advising: Yes/No
- Difficulty finding psychological support: Yes/No
- Technical difficulties: Yes/No
- Difficulty communicating with other students: Yes/No
- None of the above: Yes/No
